# Emerging roles of *N*6-methyladenosine (m^6^A) modification in breast cancer

**DOI:** 10.1186/s13578-020-00502-3

**Published:** 2020-11-25

**Authors:** Yanyan Wang, Yujie Zhang, Yushen Du, Meiqi Zhou, Yue Hu, Suzhan Zhang

**Affiliations:** 1 Department of Breast Surgery, The Second Affiliated Hospital, Zhejiang University School of Medicine, Hangzhou, 310009 Zhejiang China; 2 Department of Orthopedic Surgery, The Second Affiliated Hospital, Zhejiang University School of Medicine, Hangzhou, 310009 Zhejiang China; 3 Department of Molecular and Medical Pharmacology, University of California, Los Angeles, CA 90095 USA

**Keywords:** m^6^A, Breast cancer, Epigenetics, Molecular mechanism, Clinical applications

## Abstract

*N*6-Methyladenosine (m^6^A) is the most abundant, dynamic, and reversible epigenetic RNA modification that is found in coding and non-coding RNAs. Emerging studies have shown that m^6^A and its regulators affect multiple steps in RNA metabolism and play broad roles in various cancers. Worldwide, breast cancer is the most prevalent cancer in female. It is a very heterogeneous disease characterized by genetic and epigenetic variations in tumor cells. Increasing evidence has shown that the dysregulation of m^6^A-related effectors, as methyltransferases, demethylases, and m^6^A binding proteins, is pivotal in breast cancer pathogenesis. In this review, we have summarized the most up-to-date research on the biological functions of m^6^A modification in breast cancer and have discussed the potential clinical applications and future directions of m^6^A modification as a biomarker as well as a therapeutic target of breast cancer.

## Background

Breast cancer is the most common malignancy and leading cause of cancer-related death in women [1]. In 2018, up to 2.1 million women worldwide were diagnosed with breast cancer, occupying one out of four cancer cases among the female population [2]. At present, approximately 70–80% of non-metastatic breast cancer patients get cured, while advanced (metastatic) breast cancer patients do not attain remission using the currently available treatment regimens [1]. Breast cancer is known to be associated with molecular heterogeneity and exhibits a variety of histological features, prognostic patterns, and responses to treatment [3–5]. Thus, it is imperative to understand the underlying molecular mechanism of the development of breast cancer in detail.

Several studies have recently shown the importance of the intricate signaling at genetic, transcriptomic, and epigenetic levels that affects tumorigenesis and progression of breast cancer [6–8]. *N*6-Methyladenosine (m^6^A) is one of the most common internal epigenetic modifications found in RNA molecules [9]. After its discovery by Desrosiers in the 1970s [10], owing to the limitations in technology, research on m^6^A modification has slowly gained attention in the past couple of decades. Recently, with the advances in molecular biology and sequencing, the research on m^6^A modification has made remarkable progress [11–13]. To date, m^6^A modifications have been identified in almost every kind of RNA, including mRNA, tRNA, and non-coding RNA, and they are involved in multiple RNA processing and metabolism activities such as splicing, localization, export, translation, stabilization, and decay [14–18]. Notably, m^6^A modification sites are evolutionally conserved (mammals, insects, plants, bacteria, yeast and some viruses) and occur within a consensus sequence DRACH (D = G, A, or U; R = G or A; H = A, C, or U) [11, 12]. m^6^A methylation is not randomly distributed and is commonly detected in the coding sequences and 3′ untranslated regions (3′ UTRs), around the stop codons in mRNAs, or near the last exon in non-coding RNAs [19–21]. Deposition of m^6^A preferentially in the 5′ UTR was also observed in a few cases [22, 23].

It has become clear that the global abundance of m^6^A and expression levels of its regulators are frequently dysregulated in a variety of cancers, including breast cancer [24, 25]. The functions of m^6^A are critical for multiple biological processes such as tumor initiation, promotion, and progression in breast cancer. In this review, we first provide a comprehensive elucidation of m^6^A modification, and then focus on the emerging pathophysiological roles and molecular mechanism of m^6^A modification in breast cancer. More importantly, we highlight the potential clinical applications and future directions of m^6^A modification as a biomarker as well as a therapeutic target of breast cancer.

## Regulation of m^6^A modification

**Fig. 1:**
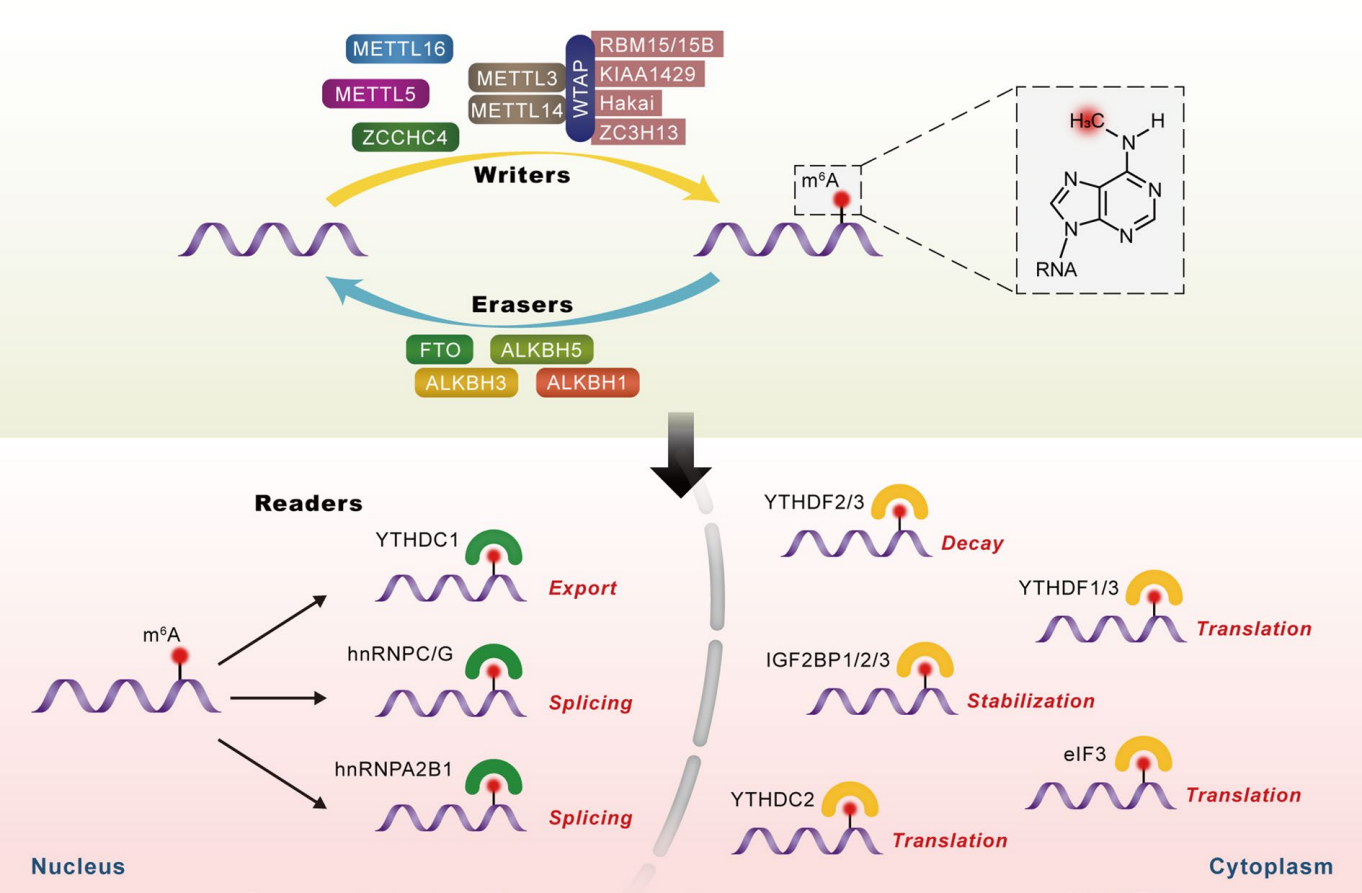
The molecular mechanism involved in m^6^A modification of consensus adenosine (A) bases. This is a dynamic and reversible epigenetic modification that is regulated by “writers” and “erasers.” m^6^A methylation is primarily catalyzed by the m^6^A methyltransferase complex comprising METTL3/METTL14/WTAP and other regulatory proteins (RBM15/15B, KIAA1429, Hakai, or ZC3H13). The erasers mainly include FTO, ALKBH5, ALKBH3, and ALKBH1. In addition to writers and erasers, “readers” are binding proteins that recognize m^6^A marks in the RNA. m^6^A modification can affect multiple steps in RNA processing, such as RNA splicing, export, translation, stabilization, and decay

The m^6^A modification, as its name suggests, involves the transfer of a methyl group to the N-6 position of the adenosine in the nucleic acid [26]. Similar to DNA and histone methylation, m^6^A modification is a dynamic and reversible biological process that is regulated by methyltransferases (also called “writers”) and demethylases (also called “erasers”). In addition to writers and erasers, “readers” are binding proteins that recognize the chemical signatures important for the regulation of m^6^A modification (Fig. 1) [27, 28].

### m^6^A writers

Writers of m^6^A methylation include the multicomponent m^6^A methyltransferase complex (MTC) comprising methyltransferase-like 3 (METTL3), METTL14, Wilms tumor 1-associated protein (WTAP), and other regulatory proteins, including RNA-binding motif protein 15 (RBM15), RBM15B, Vir-like m^6^A methyltransferase associated (VIRMA, also termed as KIAA1429 or Virilizer), Cbl proto-oncogene like 1 (CBLL1, also termed as Hakai), and zinc finger CCCH-type containing 13 (ZC3H13) [29]. In the MTC, METTL3 is the active catalyzing enzyme, while METTL14 is responsible for maintaining the catalytic activity of METTL3 and substrate recognition. The heterodimer formed by METTL3 and METTL14 is indispensable for m^6^A methylation [30, 31]. WTAP helps in binding of this METTL3/METTL14 heterodimer to regulatory proteins and in localization of MTC in nuclear spots, thereby facilitating m^6^A methylation at selective group of transcripts and regions [32]. Moreover, certain m^6^A methyltransferases do not exert their function via the MTC. METTL16, METTL5, and zinc finger CCHC-type containing 4 (ZCCHC4) are RNA m^6^A methyltransferases that directly catalyze m^6^A modification in RNA molecules [33–35].

### m^6^A erasers

Demethylases (“erasers”) are proteins that remove the m^6^A modification from RNA and include the fat mass and obesity-associated protein (FTO), α-ketoglutarate-dependent dioxygenase alk B homolog 5 (ALKBH5), ALKBH3, and ALKBH1 [36]. All these molecules belong to the α-ketoglutarate-dependent dioxygenase family of proteins and share a common mechanism for demethylation: m^6^A is oxidized to *N*6-hydroxymethyladenosine (hm^6^A) that is converted to *N*6-formyladenosine (f^6^A) before finally reverting to adenosine (A), i.e., m^6^A-hm^6^A-f^6^A-A in a step-wise manner [37]. FTO was the first m^6^A demethylase identified (2011), and it can not only remove methyl group of m^6^A in RNA, but can also demethylate N6,2-O-dimethyladenosine (m^6^A_m_), which is predominantly located in the 5′ UTR [38–40]. ALKBH5, primarily localized to the nucleus, was the second m^6^A demethylase to be identified (2013). It can remove the m^6^A modification from nuclear RNA (mostly mRNA), thereby affecting mRNA export, splicing, and stability [41, 42].

### m^6^A readers

Readers of m^6^A methylation constitute m^6^A-binding proteins that recognize the modified site and induce a series of physiological functions [43]. These proteins can be divided into three categories depending on the mechanism of m^6^A recognition: direct reader, m^6^A switch reader, and indirect reader [36]. Direct readers comprise the most-studied category and include YTH domain-containing proteins and eukaryotic translation initiation factor (eIF) 3 [36]. The YTH domain is an RNA-binding domain that interacts with m^6^A via a “tryptophan cage” [44]. There are five proteins that form the YTH domain-containing (YTHDC) family of proteins, namely, YTHDC1, YTHDC2, and YTHDF1-3 [45]. YTHDC1 and the YTHDF family are primarily localized to the nucleus and cytoplasm, respectively, while YTHDC2 is found in both the nucleus and cytoplasm [14, 46, 47]. They identify specific m^6^A sites, and accordingly regulate export, degradation as well as translation of m^6^A-containing mRNAs [48]. Heterogeneous nuclear ribonucleoproteins (hnRNPs) including hnRNPG, hnRNPC, and hnRNPA2B1 and insulin-like growth factor 2 mRNA binding proteins (IGF2BPs) including IGF2BP1, IGF2BP2, and IGF2BP3 can function as m^6^A switch readers by remodeling specific RNA structure and consequently impacting the binding mode of RNA and protein [36, 49, 50]. Fragile-X mental retardation protein (FMRP) has been recently identified to be an indirect reader since it can regulate m^6^A-modified mRNA by binding with the YTHDF proteins [51].

### m^6^A sequencing technology

m^6^A-antibody immunoprecipitation (m^6^A-IP) and methylated RNA m^6^A immunoprecipitation sequencing (MeRIP, also called m^6^A-seq) were used to reveal the landscape of transcriptome-wide m^6^A sites in 2012 [11, 12]. However, these methods could only detect m^6^A sites within 100–200 nucleotides long RNA fragments and could not identify m^6^A sites at base resolution [52]. Thus, to overcome low resolution, a series of new detection methods have been developed. For example, the RNA-antibody photo-crosslinking and immunoprecipitation (CLIP) methods (PA-m^6^A-seq, miCLIP, and UV-CLIP) are antibody-based methods with better resolution [53]. m^6^A-REF-seq or MAZTER-seq are antibody-free m^6^A-seq methods that are based on the RNA m^6^A methylation-sensitive endoribonuclease MazF. It identifies unknown m^6^A sites that have been reported to be undetectable by CLIP [54, 55]. Another antibody-free method, termed DART-seq, is based on the fusion construct of m^6^A binding protein YTH and C-to-U editing enzyme APOBEC1. This technique requires low amounts of RNA and simple library preparation [56]. It is noteworthy that the methods mentioned above mostly detect m^6^A modification indirectly and may result in inaccuracies [57]. Recently, the Oxford nanopore technology is used to study transcriptome-wide m^6^A using a direct RNA sequencing protocol, which could prevent bias associated with amplification or reverse transcription [58].

## m^6^A modification in breast cancer

With the elucidation of mechanisms involved in m^6^A modification, current research has focused on the roles of m^6^A modification in various diseases. Although studies on the function of m^6^A in breast cancer are in their early stages, increasing evidence has shown that m^6^A is essential in many aspects of this tumor, including tumorigenesis, metastasis, prognosis, and therapy resistance. Herein, we review the physiological effects of m^6^A modification in breast cancer (Table 1) and elaborate its future research trends and potential clinical applications.

**Table 1:** Roles of m^6^A regulators in breast cancer

m^6^A regulators	Role in cancer	Biological function	Target/signaling axis	Refs.
Writers				
METTL3	Oncogene	Promote cell proliferation and growth	HBXIP/let-7g/METTL3/HBXIP	[60]
		Promote proliferation and migration		[62]
		Promote cell proliferation, tumor growth; Inhibit cell apoptosis	Bcl-2	[61]
METTL14	Oncogene	Promote proliferation and migration		[62]
		Promote cell migration and invasion	Has-miR-146a-5p	[76]
		Promote cell proliferation and colony formation and inhibit cell apoptosis	LNC942-METTL14-CXCR4/CYP1B1	[71]
	Tumor suppressor	Suppress cell viability, colony formation and migratory abilities		[64]
KIAA1429	Oncogene	Promote proliferation and migration	CDK1	[65]
Hakai	Tumor suppressor	Suppress cell proliferation and migration	ERα	[79]
Erasers				
FTO	Oncogene	Promote cell proliferation, colony formation and metastasis	BNIP3	[67]
ALKBH5	Oncogene	Promote cell viability, colony formation and migratory abilities		[64]
		Increase the percentage of breast cancer stem cells	NANOG	[82]
		Promote metastasis from breast to lungs	NANOG and KLF4	[83]
	Tumor suppressor	Suppress proliferation and migration		[76]
Readers				
YTHDF3	Oncogene	Independent prognostic factor for overall survival		[25]
hnRNPC	Oncogene	Promote cell proliferation and tumor growth	dsRNA-induced interferon response	[72]
hnRNPA2/B1	Oncogene	Promote cell proliferation, decrease apoptosis, and prolong the S phase of the cell cycle	STAT3 and ERK1/2 signaling pathway	[73]
	Tumor suppressor	Suppress EMT and metastasis	PFN2	[86]
IGF2BP	Oncogene	Promote stemness of breast cancer cells	Myc	[84]
eIF3m	Oncogene	Promote the cell proliferation, migration, invasion as well as suppress apoptosis in TNBC		[71]
eIF3g	Oncogene	Promote lymph node metastasis	hnRNPU, HSZFP36 and β-actin	[87]

### Roles of m^6^A in breast cancer proliferation and apoptosis

**Fig. 2:**
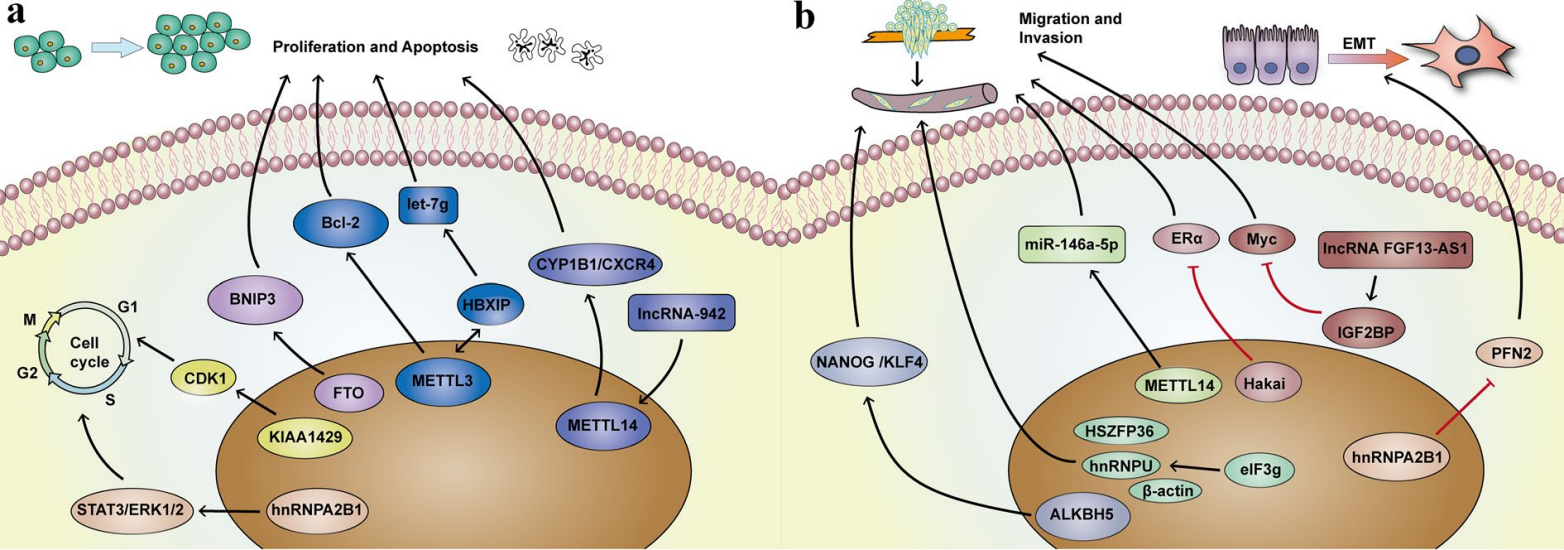
The pathophysiological roles and molecular mechanism of m^6^A modification in breast cancer. **a** m^6^A and its regulators control RNA fate and metabolism to affect proliferation, apoptosis and cell cycle. **b** The mechanism of m^6^A modification involved in breast cancer migration, invasion and metastasis

Immortality and evasion of apoptosis are the two hallmarks of cancer [59]. Numerous studies have shown the dysregulation of writers/erasers/readers associated with m^6^A are responsible for tumorigenesis and progression in breast cancer (Fig. 2a). METTL3, the core component of MTC, enhances cell proliferation via a positive feedback loop of the HBXIP/let-7g/METTL3/HBXIP axis in breast cancer [60]. METTL3 also induces proliferation, inhibits apoptosis, and accelerates tumor growth by targeting Bcl-2 [61]. Overexpression of the METTL3/14 m^6^A methylation complex results in malignant transformation [62]. METTL14 interacts with and modifies lncRNA-942 by adding m^6^A to enhance the expression and stability of CYP1B1 and CXCR4, respectively, thereby increasing cell proliferation and colony formation and suppressing cell apoptosis [63]. Interestingly, a similar study showed that the overexpression of METTL14 inhibits cell viability and colony formation in breast cancer [64]. KIAA1429 is an oncogene and it promotes breast cancer cell proliferation and colony formation by stabilizing the *CDK1* mRNA (a cell cycle regulator) [65]. Similarly, Lewinska et al. [66] demonstrated that decrease in the m^6^A signature promotes cell cycle arrest and senescence, thereby exerting anticancer effects.

As an m^6^A eraser, FTO demethylates the 3′ UTR of the *BNIP3* mRNA and induces its decay in an YTHDF2-independent manner, resulting in breast cancer cell proliferation, colony formation, and metastasis [67]. Polymorphisms in FTO are associated with breast cancer, especially estrogen receptor (ER)-positive breast cancer [68, 69]. Estrogen stimulates breast cancer cell proliferation by upregulating FTO and activating PI3K/Akt signaling [69]. Moreover, a recent study has demonstrated that FTO mediates the survival of metabolically adaptable triple-negative breast cancer (TNBC) cells in glutamine-deficient microenvironments [70]. The function of ALKBH5 in breast cancer is controversial. Wu et al. [64] have shown that silencing ALKBH5 leads to inhibition of breast cancer cell viability, colony formation, and migration. However, Fry et al. demonstrated the overexpression of ALKBH5 and METTL3/14 in immortalized human mammary epithelial cells. Depletion of ALKBH5 increases cell proliferation and migration [62].

The expression of eIF3m, one of the 13 subunits of m^6^A reader eIF3, positively correlates with the development and progression of breast cancer. Downregulation of eIF3m inhibits breast cancer proliferation and increases the rate of apoptosis [71]. Wu et al. have reported high levels of hnRNPC associated with breast cancer proliferation. Downregulation of hnRNPC promotes the formation of endogenous double-stranded RNA and induces immune response that results in antiproliferative activity [72]. hnRNPA2B1 also has a positive role in breast cancer. Knockdown of hnRNPA2B1 decreases breast cancer cell proliferation, increases apoptosis, and prolongs the S phase of cells by inhibiting STAT3/ERK1/2 signaling [73].

### Roles of m^6^A in breast cancer migration, invasion and metastasis

Metastasis is a major cause of cancer-related deaths. Although the survival rate of breast cancer has improved immensely over the past decades, the therapeutic effect of metastatic breast cancer is still not optimistic [74]. Migration and invasion of tumor cells are key processes in cancer metastasis (Fig. 2b) [75]. METTL14 promotes the migration and invasion of breast cancer cells by directly regulating hsa-miR-146a-5p and m^6^A modification [76]. Similarly, KIAA1429 has also been found to promote breast cancer cell migration and invasion [65]. ERs constitute the most active transcription factors involved in breast cancer. Inhibiting ERα activity is currently used as a strategy for treating patients with ER-positive breast cancer [77, 78]. Hakai is a coregulator of ERα and suppresses breast cancer cell migration by competitively binding to ERα [79]. Although breast cancer stem cells (BCSCs) constitute a minor proportion of breast cancer cells, accumulating evidence has demonstrated the vital role of BCSCs in tumor initiation, progression, and metastasis [80, 81]. Hypoxia stimulates ALKBH5 or ZNF217 that stabilize the *NANOG* and *KLF4* mRNAs and induce the phenotype associated with BCSCs and lung metastasis [82, 83]. IGF2BP binds to lncRNA FGF13-AS1 and Myc to form a positive feedback loop to regulate breast cancer cell stemness [84]. Epithelial–mesenchymal transition (EMT) accelerates the progress of tumor metastasis [85]. Liu et al. [86] demonstrated that hnRNPA2B1 inhibits EMT and metastasis in breast cancer by directly binding to *PFN2* mRNA and reducing its stability. Conversely, eIF3m promotes breast cancer cell migration and invasion by activating EMT [71]. eIF3g, another subunit of eIF3, interacts with hnRNPU, HSZFP36, and β-actin in the nucleus and promotes the metastasis of breast cancer to the lymph nodes [87].

### Roles of m^6^A in the clinicopathology and prognosis of breast cancer

**Fig. 3:**
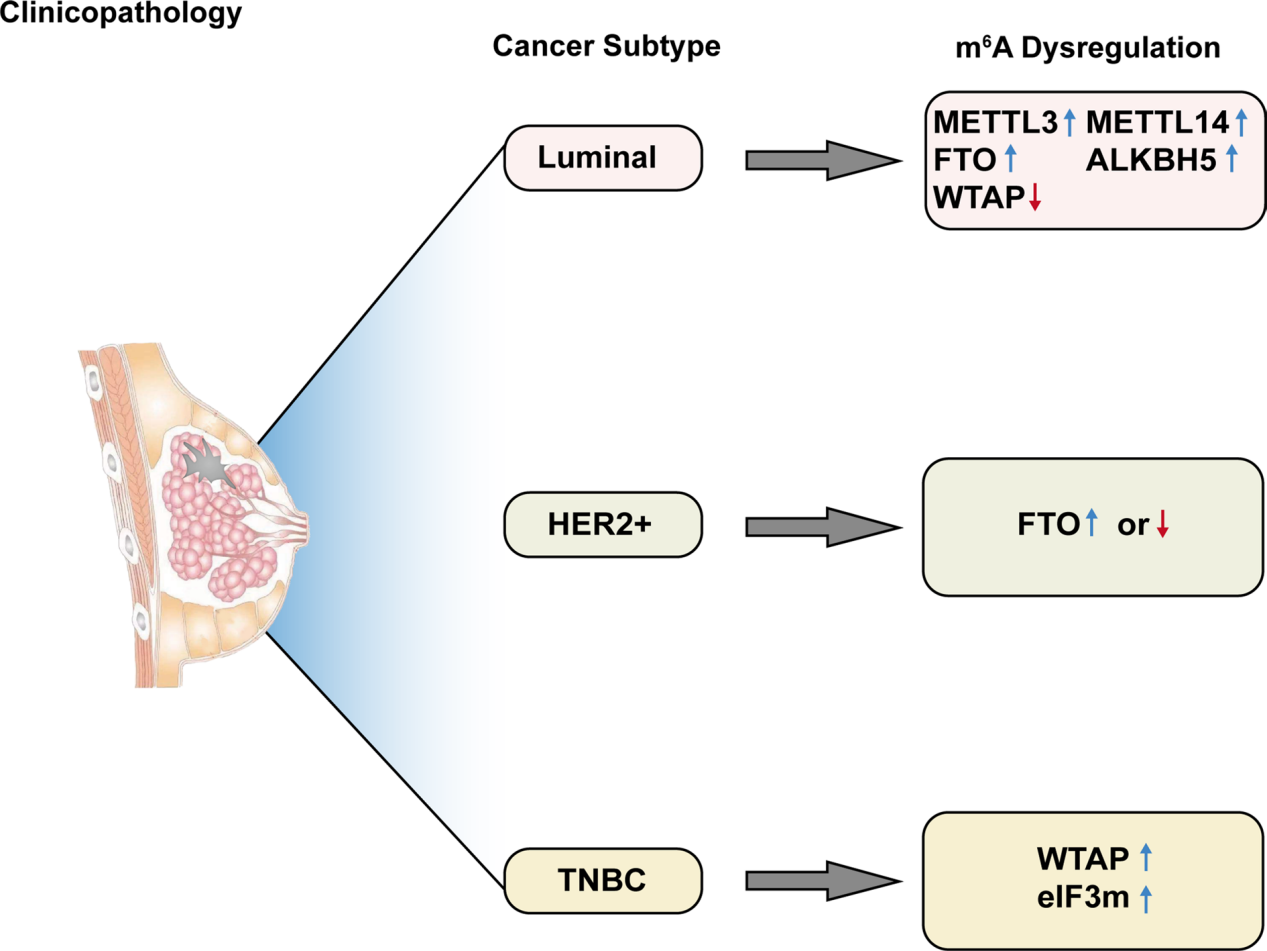
The roles of m^6^A modification in the clinicopathology of breast cancer

A growing number of studies have confirmed the correlation between m^6^A modification and clinical pathological characteristics and prognosis of breast cancer (Fig. 3). Typically, breast cancer is classified into three major subtypes based on molecular markers: ER or progesterone receptor (PR)-positive (luminal A and luminal B), human epidermal growth factor receptor 2 (HER2)-positive, and TNBC [3, 88]. Different subtypes of breast cancer are associated with distinct etiologies, response to treatment, and prognosis. Wu et al. [64] reported that METTL3, METTL14, FTO, and ALKBH5 are upregulated and WTAP is downregulated in luminal breast cancer patients, while the expression level of FTO is significantly decreased in HER2-positive breast cancer. However, the study by Tan et al. demonstrated overexpression of FTO in hormone receptor-negative and HER2-positive breast cancer. A significant proportion of FTO-positive cells have also been reported in P53-positive or histological grade 3 breast cancer [89]. Overexpression of eIF3m has been observed in TNBC but not in non-TNBC or normal breast tissues and it reduces overall survival (OS), relapse-free survival, and post-progression survival in breast cancer patients [71]. Using the data from The Cancer Genome Atlas-Breast Cancer cohort, Liu et al. revealed that the overexpression of YTHDF1, YTHDF3, and KIAA1429 is predictive of poor prognosis. Especially, YTHDF3 is an independent prognostic factor of OS in breast cancer patients [25].

Zeng et al. performed a case–control study based on Chinese population to determine the correlation between polymorphisms in FTO and risk associated with prognosis of breast cancer patients. Their results showed variants of FTO are concerned with varying susceptibility of breast cancer; however, they cannot predict survival outcomes in patients with this disease [90]. Meanwhile, it is acknowledged that obesity increases the risk of breast cancer substantially, but the molecular mechanism involved remain to be understood [91]. As the name implies, FTO is intimately associated with obesity. Thus, the advent of FTO may well explain the relationship between obesity and breast cancer [69, 92]. In addition, epidemiological studies have found that reproductive history is linked to the development of breast cancer. The risk of breast cancer is significantly less in early pregnancy (before age 20), while the risk transiently increases after parturition [91, 93]. Peri et al. [94] have demonstrated that hnRPA2B1 is overexpressed in the mammary tissues of post-menopausal parous women, suggesting that m^6^A modification may contribute to the correlation between pregnancy and breast cancer.

## Discussion

With the discovery of FTO as an m^6^A demethylase, research on m^6^A modification has become the hotspot of epigenetics. Recent reports have demonstrated that m^6^A-related regulators play essential and diverse biological functions in the development of various types of cancer, including breast cancer, glioblastoma, hepatocellular carcinoma, acute myeloid leukemia, and cervical cancer [24, 95–97]. This review summarizes the recent advances in the understanding of the roles, mechanisms, and potential clinical applications of m^6^A in breast cancer. Notably, the specific mechanism for m^6^A modification in breast cancer is complex and even inconsistent among studies. For instance, Wu et al. [64] showed that m^6^A methylation suppresses the growth and metastasis of breast cancer, while Fry et al. [62] reported malignant progression with increasing m^6^A methylation. This “double-edged sword” phenomenon is also reported in other tumors [98] and may be attributed to differences in the origin of tumor tissues, intratumoral heterogeneity, and ethnicity at the macro level. For example, the polymorphisms rs9939609 and rs1477196 in FTO are implicated in an increased risk of breast cancer among women excluding those from Iran [99]. Moreover, at the molecular level, there are two types of m^6^A sites in different cell lines: structural m^6^A sites and dynamic m^6^A sites. Dynamic m^6^A sites are cell-specific sites regulated by spatio-temporal regulators [100]. This category of m^6^A sites can make the gene play diverse roles in different cells that may contribute to the phenomenon.

Studies have shown the importance of m^6^A regulatory enzymes as novel potential biomarkers for the early diagnosis and prognosis of breast cancer. Different enzymes involved in catalyzing m^6^A modification correlate with specific molecular subtypes of breast cancer that are classified based on the presence of certain biomarkers (ER, PR, and HER2). For example, eIF3m is overexpressed in TNBC, while it is expressed to the same extent in tumors and corresponding adjacent normal breast tissues in non-TNBC. The upregulation of eIF3m represents poor pathological differentiation, high degree of malignant transformation, and increased rates of lymph node and distant metastases in TNBC. Moreover, elevated expression of eIF3m implies poor survival outcomes for TNBC patients [71]. Therefore, eIF3m may be a reliable biomarker of TNBC. Of interest, we also found that both the m^6^A writer and eraser genes are aberrantly overexpressed and play oncogenic roles in breast cancer. Thus, global m^6^A signatures may be unreliable as diagnostic and prognostic biomarkers in patients with breast cancer. To that extent, the m^6^A profiles of specific transcripts or transcript loci could serve as better biomarkers. However, the techniques currently available for studying transcriptome-wide m^6^A modification are not precise enough [52]. This has resulted in the difficulty in fully understanding the correlation between m^6^A-modified RNAs and disease. Additionally, these methods are limited by the requirement of large amounts of RNA, experienced technical skills, and high cost, thereby limiting the feasibility of m^6^A-seq in large-scale screening [57]. Therefore, novel detection methods with high precision, reduced sample volume, and low cost are warranted. This will help develop m^6^A profiles/signatures of specific transcripts or transcript loci as early diagnostic and prognostic biomarkers for breast cancer. The improved methods of m^6^A-seq may enable the use of peripheral blood for screening of cancer in the future.

m^6^A may also serve as a novel therapeutic target in breast cancer. Targeting dysregulated m^6^A regulators represents an attractive strategy for cancer therapy. However, only a few studies have focused on the development of potent and specific drugs that target m^6^A regulators in breast cancer. MO-I-500 is a small-molecule inhibitor of the m^6^A demethylase activity of FTO and inhibits the survival and/or colony formation of a SUM149 triple-negative inflammatory breast cancer cell line [70]. In addition to small-molecule compounds, PROTAC (proteolysis targeting chimera)-based inhibitors can also be developed to treat breast cancer by selectively degrading dysregulated m^6^A regulators [101]. Systemic therapies, such as chemotherapy, radiotherapy, endocrine therapy, and targeted therapy, comprise the most important arm of breast cancer treatment [1]. Resistance to these therapies is catastrophic and contributes to failed treatment and/or cancer recurrence [102, 103]. Recent studies have indicated that dysregulation of m^6^A regulators plays an important role in developing resistance to therapy in cancer [104, 105]. Klinge et al. observed higher RNA and protein levels of hnRNPA2B1 in tamoxifen-resistant breast cancer cells. The upregulation of hnRNPA2B1 alters the expression of multiple miRNAs and reduces the sensitivity of MCF-7 cells to tamoxifen [106], suggesting the importance of hnRNPA2B1 in resistance to endocrine therapy. Future research should focus on abrogating m^6^A-mediated resistance of breast cancer cells via different treatment regimens.

Immunotherapy is emerging as a new treatment modality in breast cancer, especially metastatic breast cancer [107]. Owing to the unsatisfactory effect of immunotherapy in the early stages of patients with breast cancer, breast cancer has previously been assumed to be unresponsive to the immunotherapy [108]. This could be attributed to the lacunae in the molecular mechanism in breast cancer that has resulted in the slow development of effective immunotherapy in such patients. Recent studies have shown the regulatory effect of m^6^A RNA modification on host immunity and in enhancing anticancer immunotherapy. Depleting FTO promotes the degradation of downstream genes PD-1, CXCR4, and SOX10 in an m^6^A-dependent manner, thereby sensitizing patients with melanoma to anti-PD-1 checkpoint blockade therapy [109]. Similarly, Han et al. demonstrated a new mechanism for immune evasion: the m^6^A reader YTHDF1 binds to and promotes the translation of mRNAs encoding lysosomal proteases that result in the reduction of cross-presentation of tumor antigens in dendritic cells. Silencing YTHDF1 inhibits immune evasion and improves the efficacy of anti-PD-1 therapy [110]. Given the vital roles of m^6^A modification in breast cancer as well as the promising effect of immunotherapy in other tumors, combining m^6^A signatures and anticancer immunotherapy may serve as a breakthrough in breast cancer immunotherapy.

Currently, the roles and mechanisms involved in m^6^A modification in breast cancer remain to be elucidated and several issues need to be addressed in the future. First, high-throughput research on m^6^A modification should be performed to generate m^6^A methylation-centric networks in breast cancer. Second, although researchers have noted the potential of m^6^A as a diagnostic and prognostic marker for breast cancer, no studies that have focused on the sensitivity or specificity of this marker in large patient cohorts. Current m^6^A sequencing technologies are not sufficient to support large-scale screening. Thus, a novel sequencing technology is indispensable to study the role of m^6^A in breast cancer. Third, there is preliminary evidence for the potential of m^6^A as a therapeutic target for breast cancer. Studies have only focused on the molecular mechanisms involved at this stage and a few reports have focused on drug development and pre-clinical/clinical trials. Future experiments should examine the efficacy of m^6^A-targeted drugs alone or in combination with other treatments for breast cancer.

## Conclusions

Taken together, we have discussed the dysregulation of m^6^A modification in breast cancer to help develop broad clinical applications in the prevention, treatment, and management of breast cancer. Detailed efforts to understand the underlying mechanism of m^6^A modification in breast cancer, identify and develop diagnostic and prognostic factors, and devise m^6^A-targeted therapy will help better treat patients with breast cancer in the future. This will also highlight the diverse (undiscovered) aspects of m^6^A modification and mark the beginning of the era of RNA epigenetics in cancer therapy.

## Data Availability

Not applicable.

## Abbreviations

ALKBH5: α-Ketoglutarate-dependent dioxygenase alk B homolog
BCSC: Breast cancer stem cell
CBLL1: Cbl proto-oncogene like 1
CLIP: RNA-antibody photo-crosslinking and immunoprecipitation
eIF3: Eukaryotic translation initiation factor 3
EMT: Epithelial–mesenchymal transition
ER: Estrogen receptor
f^6^A: *N*6-Formyladenosine
FMRP: Fragile-X mental retardation protein
FTO: Fat mass and obesity-associated protein
HER2: Human epidermal growth factor receptor 2
hm^6^A: *N*6-Hydroxymethyladenosine
hnRNP: Heterogeneous nuclear ribonucleoprotein
IGF2BP: Insulin-like growth factor 2 mRNA binding protein
m^6^A: *N*6-Methyladenosine
m^6^A-IP: M^6^A-antibody immunoprecipitation
MeRIP-seq: Methylated RNA immunoprecipitation sequencing
METTL: Methyltransferase like
MTC: Methyltransferase complex
OS: Overall survival
PR: Progesterone receptor
PROTAC: Proteolysis targeting chimera
RBM: RNA-binding motif
TNBC: Triple-negative breast cancer
UTR: Untranslated region
VIRMA: Vir-like m^6^A methyltransferase associated
WTAP: Wilms tumor 1-associated protein
YTHDC: YTH domain-containing
ZC3H13: Zinc finger CCCH-type containing 13
ZCCHC4: Zinc finger CCHC-type containing 4
